# Effects of sleep disturbance, cancer-related fatigue, and psychological distress on breast cancer patients’ quality of life: a prospective longitudinal observational study

**DOI:** 10.1038/s41598-024-59214-0

**Published:** 2024-04-15

**Authors:** Lin Tao, Jieying Lv, Ting Zhong, Xiaohong Zeng, Manxia Han, Lan Fu, Hong Chen

**Affiliations:** 1 Cancer Day-Care Unit, Department of Medical Oncology, Cancer Center, West China Hospital, Sichuan University/West China School of Nursing, Sichuan University, West China Hospital, Sichuan University, Chengdu, 610041 China; 2 Division of Head & Neck Tumor Multimodality Treatment, Cancer Center, West China Hospital, Sichuan University/West China School of Nursing, Sichuan University, West China Hospital, Sichuan University, Chengdu, 610041 China; 3 Department of Nursing, West China Hospital, Sichuan University/ West China School of Nursing, Sichuan University, West China Hospital, Sichuan University, No. 37, Guoxuexiang, Wuhou District, Chengdu, 610041 China

**Keywords:** Breast cancer, Sleep disturbance, Cancer-related fatigue, Psychological distress, Quality of life, Latent growth model, Cancer, Psychology, Health care, Oncology, Risk factors

## Abstract

More attention has gone to researching the cancer-related fatigue (CRF)–sleep disturbance (SD)–psychological distress (PD) symptom cluster in breast cancer patients during the chemotherapy period, but the change trend and heterogeneous development track in the whole treatment stage remain unclear, and it is also unclear whether the appearance of and changes in one symptom cause changes in other symptoms and quality of life (QoL). This study, using breast cancer patients’ data collected through a validated questionnaire, examined the relationships between SD, CRF, PD, and QoL using latent growth modeling analyses. CRF developmental trajectories showed an upward trend over five surveys (slope = 0.649, *P* < 0.001); PD showed a significant weakening trend (slope = − 0.583, *P* < 0.001); SD showed an increasing trend (slope = 0.345, *P* < 0.001), and QoL showed a statistically significant weakening trend (slope = − 0.373, *P* < 0.001). The initial CRF (coefficient = − 0.233, *P* < 0.01), PD (coefficient = − 0.296, *P* < 0.01), and SD (coefficient = − 0.388, *P* < 0.001) levels had a statistically significant negative effect on initial QoL level. The linear development rate of PD was statistically significant and negatively affected that of QoL (coefficient = − 0.305, *P* < 0.05), whereas the quadratic development rate of SD negatively affected that of QoL (coefficient = − 0.391, *P* < 0.05). Medical staff should identify the change characteristics of different variables based on SD, CRF, PD, and QoL change trajectories, and advance the intervention time, as changes in variables affect other variables’ subsequent changes.

## Introduction

Breast cancer is one of the most common cancers among women. According to the global cancer statistics (GLOBOCAN) in 2020, the number of new breast cancer cases has reached 2.26 million, surpassing lung cancer and ranking first among the world's malignant tumors^1^. With continuous improvements in diagnosis and treatment protocols, breast cancer patients’ survival time has been prolonged, but their quality of life (QoL) has not significantly improved^2,3^, possibly due to adverse reactions to treatment. Studies have shown that cancer patients may report more than 10 symptoms during their disease diagnosis, treatment, and recovery process^4^. These symptoms interact with each other, seriously affecting patients’ physical and mental health and significantly reducing their QoL.

Currently, cancer symptom management has evolved from managing a single symptom to managing multiple related symptoms within a symptom cluster. Williams^5^ believes that there are three ways in which symptoms within a symptom cluster are interrelated: first, they have a common mechanism of occurrence; and second, one sentinel symptom leads to the development or worsening of other symptoms; third, the side effects of treating one symptom cause other symptoms. Understanding the interrelatedness of symptoms within a symptom cluster can help clinicians develop optimal symptom cluster management strategies. For example, in the pain, cancer-related fatigue (CRF), and sleep disturbance (SD) symptom cluster, pain can affect patients' sleep quality, leading to CRF, so treating pain can alleviate the symptom cluster^6^. Wood et al.^7^ found that inflammatory responses can cause the simultaneous occurrence of symptoms such as CRF, SD, lack of appetite, and low mood, so interventions to reduce inflammatory responses can alleviate the symptom cluster. Therefore, research on the mechanisms of interaction between symptoms can make it possible for one intervention to effectively manage multiple symptoms within a symptom cluster, thereby simplifying patients' self-management and promoting healthy outcomes.

The CRF-depression-SD symptom cluster is among those frequently reported by breast cancer patients^8^. A study investigated 372 patients with breast cancer who received chemotherapy. Of these, 99.2% felt tired, 87.4% did not sleep enough, and 93.3% had depressive symptoms^9^. The proportion of patients with 0, 1, 2, and 3 symptoms was 0.8%, 3.0%, 11.8%, and 84.4%, respectively. Spearman correlation analysis showed that CRF, SD, and depression were significantly related, and that each one was negatively related to QoL. Another longitudinal study on breast cancer patients investigated CRF, depression, and SD at three timepoints (before, after, and 6–8 months) after chemotherapy. The results showed that the three symptoms were related at all three timepoints, and symptom severity at previous timepoints could predict the severity of the same symptom at subsequent timepoints^10^. CRF incidence showed no significant difference between different cycles of chemotherapy, but CRF severity showed a clear pattern in each cycle. During the first 5–6 days of each cycle, patients experienced moderate to severe CRF levels, but it gradually decreased to mild levels within 14 days^11,12^. Considering the high incidence of the CR-depression-SD symptom cluster in breast cancer patients, medical staff need to identify its occurrence mechanism in time and develop corresponding measures to reduce patients’ symptom burden.

In addition, many studies have shown that psychosocial symptoms, especially anxiety and depression, persist throughout breast cancer treatment^13,14^ and occur in clusters^15,16^. In the literature, the term psychological distress (PD) is often used as a general term for anxiety and/or depressive symptoms^17^. PD extends along a continuity, ranging from a normal reaction to a diffusion situation to potentially disabling problems^18^. Bjerkeset et al.^19^ found that CRF and PD often occur within clusters, with 13% of women experiencing both CRF and high PD levels.

At present, more attention has gone to researching the CRF-SD-PD symptom cluster in breast cancer patients during the chemotherapy period, but the change trend and heterogeneous development track in the whole treatment stage remain unclear. Although some studies have explored this symptom group using regression analysis, correlation analysis, and path analysis^9,10,19^ and proposed that there may be a linear growth relationship between the three, the interaction mechanism between them and their impact on QoL cannot be explained in depth. Avis et al.^20^ identified seven symptom subgroups with pain, CRF, SD, and psychological symptoms as the core using the hidden Markov model, and preliminarily explored the relationship between different subgroups and QoL, but the interrelationship between symptoms remains unclear. For example, it is unclear whether the appearance of and changes in one symptom cause changes in other symptoms. Through cross lagged regression analysis and parallel latent variable growth models, it is helpful to comprehensively examine the dynamic development relationship, trajectory characteristics, and interaction mechanism between CRF, SD, PD, and QoL. Therefore, this study will analyze how patterns of CRF, SD, and PD predict QoL from diagnosis to after chemotherapy through cross-lagged regression analysis and parallel latent variable growth models.

## Methods

### Design and setting

This prospective longitudinal observational study ranged from 15 to 22 months, with a mean follow-up period of 18 months (SD = 2.26). It was conducted in five Grade III A (> 500 beds) comprehensive hospitals in Sichuan Province, China, from May 2019 to March 2021.

### Ethical considerations and consent to participate

This study was approved by the authors’ institution; written informed consent was obtained from all the patients for research purposes. The Clinical Trial and Biomedical Ethics Committee of West China Hospital, Sichuan University (No. 2020(564)) approved the data collection procedures involving the study participants to ensure that they were conducted in accordance with ethical standards.

### Participants

This study selected patients diagnosed with breast cancer in the target hospitals’ breast surgery departments. *Inclusion criteria* female patients, 18–65 years old, diagnosed with breast cancer by pathological biopsy and planning to undergo surgery, estimated survival time > 12 months, normal mental and cognitive functions, and can cooperate to complete treatment, follow-up, and the questionnaire survey. *Exclusion criteria* recurrence of breast cancer, distant metastasis at the time of diagnosis, concurrently suffering from other cancers, pregnant, adjuvant chemotherapy confirmed as not required by the doctor in charge after the post-operative pathology report, local or distant metastasis at follow-up, adjuvant chemotherapy not completed in the same hospital (so the exact treatment time could not be assessed), and phone not answered by the participants within three consecutive tries at follow-up.

### Procedures

The research team included breast surgery and oncology physicians and nurses. The researchers obtained the consent and support of the relevant departments of the five hospitals, then contacted the follow-up nurses in the breast surgery and breast oncology departments of each hospital, and finally conducted online training. The researchers maintained real-time contact with the follow-up nurses in each hospital through WeChat, to address any issues that arose during follow-up. Each study participant completed their surgery and chemotherapy at the same hospital. When physicians found breast cancer cases that met the recruitment criteria in the first outpatient clinic, they obtained patients’ informed consent, and then included them in the research group for follow-up management. After the patients were enrolled, the follow-up nurses established a follow-up database and were responsible for tracking the diagnosis, operation, date of chemotherapy, and chemotherapy plan, and they sent this data to the researchers, who made a follow-up plan. Data collection was mainly conducted by follow-up nurses in each hospital. After data collection at each stage was completed, the original data were packaged and sent to the researchers by the follow-up nurses for unified management. The researchers participated in quality control, regularly checked and adjusted the follow-up plan, and urged the follow-up nurses to complete the follow-up on time.

Five timepoints were selected for longitudinal tracking of patients’ SD, CRF, PD, and QoL: within one week after initial cancer diagnosis (T1), within one week after the start of surgical treatment (T2), within one week after the end of all courses of chemotherapy (T3), six months after the end of chemotherapy (T4), and 12 months after the end of chemotherapy (T5). The researchers screened patients based on their outpatient medical records; conducted one-on-one, face-to-face, semi-structured interviews with those who met the criteria; and informed them of the study’s purpose and follow-up arrangements. After patients were fully informed and had agreed to participate in this study, baseline data (T1) were collected: socio-demographic characteristics, disease-related data, SD, CRF, PD, and QoL. After the patients were discharged, dedicated follow-up phone calls were made during T2–T5, either at 09:00–11:00 or 16:00–18:00 h, to investigate the patients’ SD, CRF, PD, and QoL status. To reduce loss at follow-up, small gifts were distributed during the follow-up period, and each patient received a free physical examination. This study included as large a sample size as possible.

### Measures

#### Sleep disturbance

The Pittsburgh sleep quality Index (PSQI), developed by Buysse^21^, was used to measure SD. The scale consists of 19 self-evaluated items and 5 other-evaluated items, of which only the first 18 self-evaluated items are scored (0–3 points) across seven dimensions: sleep quality, sleep latency, sleep duration, habitual sleep efficiency, SDs, use of sleep medicine, and daytime dysfunction. The total score across dimensions constitutes the total PSQI score (0–21 points). The higher the score, the worse the sleep quality; a total score > 7 indicates SD, while ≤ 7 indicates good sleep. The Cronbach's alpha values for the scale in this study is 0.89.

#### Cancer-related fatigue

The Chinese version of the Multidimensional Fatigue Symptom Inventory-Short Form (MFSI-SF) was used to measure CRF^22^. This is a self-report assessment composed of 27 items and five subscales: general fatigue, physical fatigue, emotional fatigue, mental fatigue, and vigor. For the four fatigue subscales, each item is assessed on a 5-point scale from 0 (not at all) to 4 (extremely serious); whereas for vigor, it is assessed from 0 (nearly always) to 4 (never). The total score is thus 0–108, with higher scores indicating more CRF. This instrument has excellent psychometric properties in the Chinese context^22^. The Cronbach's alpha values for the scale in this study is 93.

#### Psychological distress

The Distress Thermometer recommended by the National Comprehensive Cancer Network^23^ was used to measure patients’ PD. It is a self-evaluation tool, scored from 0 (no distress) to 10 (extreme distress). A Distress Thermometer score ≥ 4 indicates moderate to severe PD; < 4 indicates normal mood swings, with no special intervention required. Zhang et al.^24^ found that the Distress Thermometer had excellent psychometric properties for China.

#### Quality of life

QoL was measured using the Functional Assessment of Cancer Therapy-Breast (FACT-B)^25^, composed of 36 items and five subscales: physiological, social/family, emotional, functional status, and additional concerns about breast cancer. Items are scored on a 5-point scale from 0 (not true at all) to 4 (true nearly all the time), with total scores from 0 to 144; higher scores indicate better QoL. Cronbach’s alpha for the FACT-B was 0.951.

### Data analysis

SPSS version 26.0 and Mplus version 8.3 were used for data management and analysis. Continuous variables were described as mean ± standard deviation, and categorical variables were described by frequency and percentage. As the dimensions of the four research variables (CRF, SD, PD, and QoL) were not uniform, each variable data were merged at five timepoints, and then standardized and preprocessed. A smoothed line graph was used to visualize the change trajectories of the four variables across different timepoints. Harman’s one-factor test was used to test for common method bias, Pearson correlation analysis was used to investigate relationships between the variables at different timepoints, Mplus version 8.3 was used to construct linear growth, and quadratic growth models to explore the variables’ development trends^26^. A parallel impact model was established to explore the effects of the intercept and trajectory of change of CRF, SD, and PD on the intercept, and trajectory of change of the QoL. Finally, a cross-lagged panel model was established using Mplus to explore the cross-lagged and time-series effects of SD, CRF, and PD on QoL. The significance level was set at 0.05.

## Results

### Participant details

**Figure 1:**
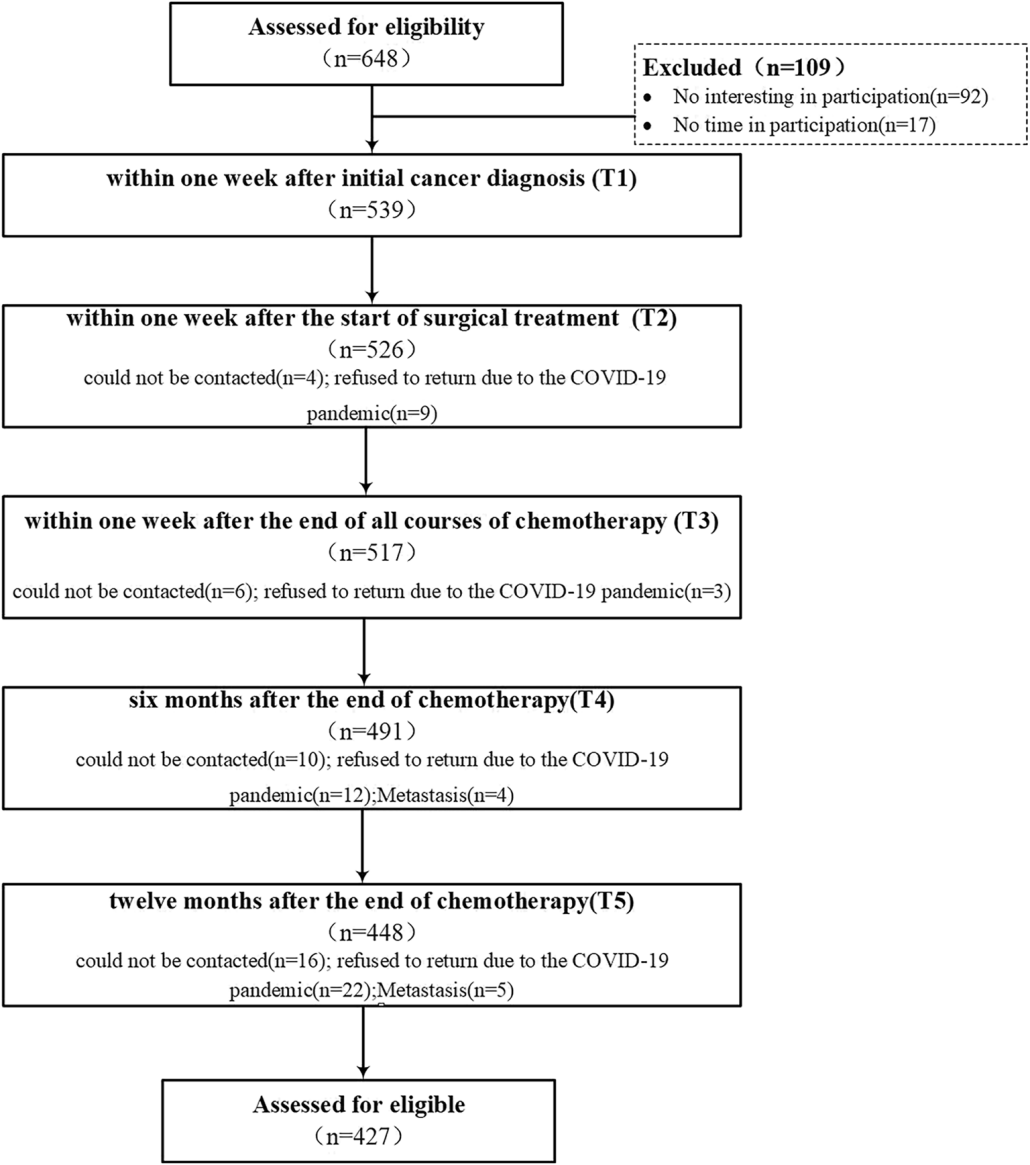
Study flow diagram.

While 648 patients were initially assessed for eligibility, after excluding 109 who did not meet the inclusion criteria, 539 patients were assessed at T1. Subsequently, during T2, T3, T4, and T5 visits, 526, 517, 491, and 448 patients, respectively, were analyzed. Thereafter, as 21 patients were excluded from the analysis, 112 total samples were lost; thus, 427 patients were assessed (Fig. 1).

A difference test was performed between all data—both lost and valid—for the following variables: age (χ^2^ = 2.087, *P* = 0.554), education (χ^2^ = 1.258, *P* = 0.739), stage of tumor (χ^2^ = 1.640, *P* = 0.441), whether to keep the breasts (χ^2^ = 0.175, *P* = 0.675), employment (χ^2^ = 0.353, *P* = 0.838), having or not having minor children (χ^2^ = 0.457, *P* = 0.499), type of health coverage (χ^2^ = 0.472, *P* = 0.790), and monthly family income per capita (χ^2^ = 0.682, *P* = 0.711). There were no significant differences, indicating no structured loss among this study’s participants. The results are detailed in Table 1.

**Table 1:** Demographic and clinical characteristics.

Variables	Attributes	Lost data samples				Total	χ2	*P*
		Follow-up group		Group that missed follow-ups				
		n	%	n	%			
Age (years)	≤ 39	96	22.5	23	20.5	119	2.087	0.554
	40–49	149	34.9	47	42.0	196		
	50–59	123	28.8	27	24.1	150		
	≥ 60	59	13.8	15	13.4	74		
Education	Primary school	73	17.1	15	13.4	88	1.258	0.739
	Middle school	93	21.8	28	25.0	121		
	Senior school	142	33.3	39	34.8	181		
	College or above	119	27.9	30	26.8	149		
Marital status	Married	262	61.4	68	60.7	330	0.657	0.720
	Unmarried	71	16.6	16	14.3	87		
	Divorced	94	22.0	28	25.0	122		
Stage of tumor	I	93	21.8	27	24.1	120	1.640	0.441
	II	208	48.7	47	42.0	255		
	III	126	29.5	38	33.9	164		
Whether to keep the breasts	Yes	136	31.9	38	33.9	174	0.175	0.675
	No	291	68.1	74	66.1	365		
Employment	Full-time job	315	73.8	80	71.4	395	0.353	0.838
	Part-time job	49	11.5	13	11.6	62		
	Unemployed	63	14.8	19	17.0	82		
Having or not having minor children	Yes	153	35.8	44	39.3	197	0.457	0.499
	No	274	64.2	68	60.7	342		
Type of health coverage	Self-pay	50	11.7	11	9.8	61	0.472	0.790
	Public fee	66	15.5	16	14.3	82		
	Medical insurance	311	72.8	85	75.9	396		
Monthly family income per capita (USD)	< 450	186	43.6	44	39.3	230	0.682	0.711
	450–750	144	33.7	40	35.7	184		
	> 750	97	22.7	28	25.0	125		

### Description of variables and analysis of development trajectory

**Figure 2:**
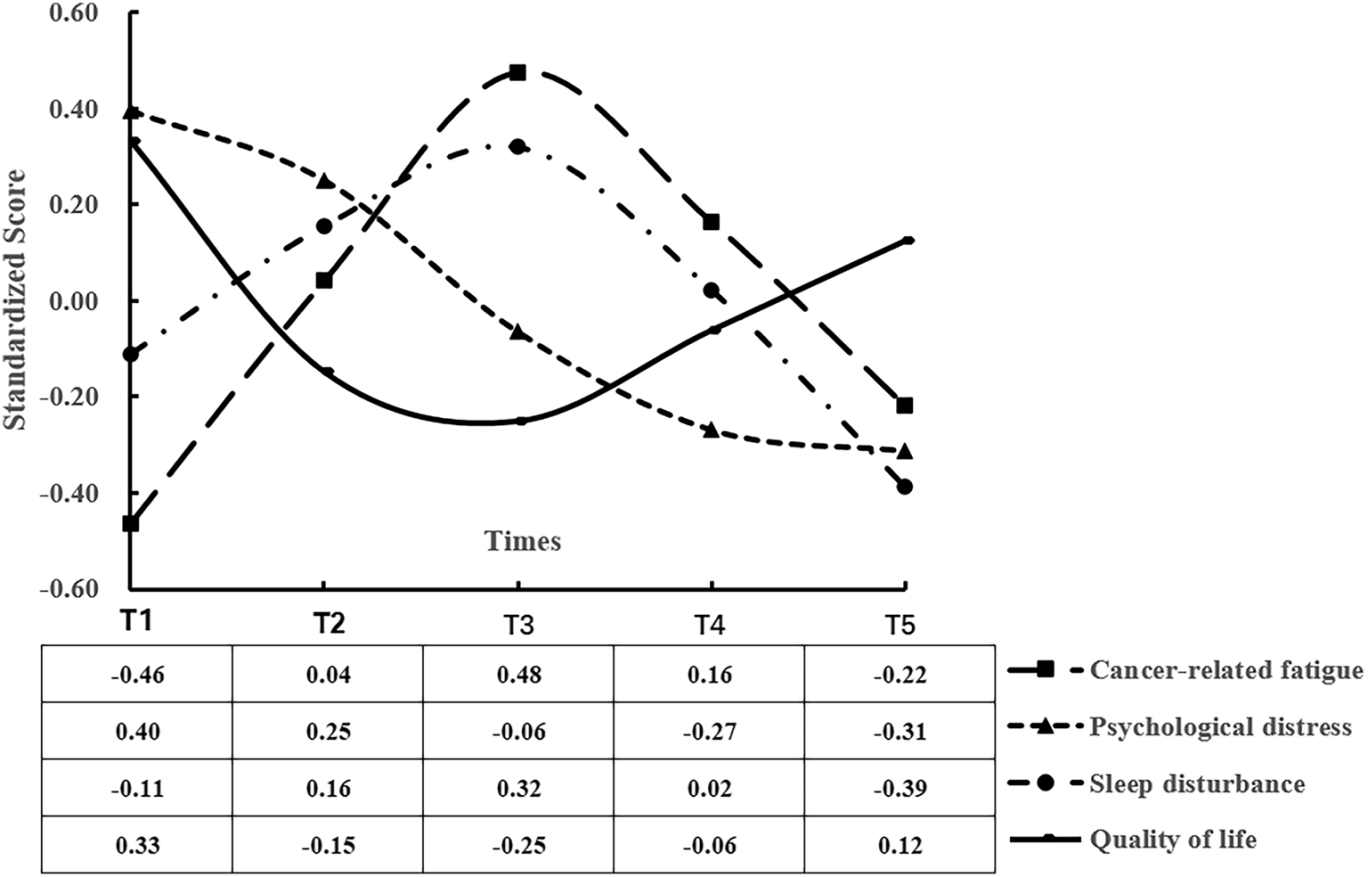
Variables’ development trajectory diagram.

Figure 2 shows the average scores for SD, CRF, PD, and QoL at different measurement timepoints, during which each variable showed normal distributions at 0.734 (< 3) and 1.174 (< 10) for maximum absolute kurtosis and skewness, respectively^27^, thus facilitating subsequent analyses.

### Correlation analysis

Correlation analysis was used to explore the correlation between the four variables (Table 2). The five measurements of each variable all showed a certain stability. The correlation coefficient between measurements was in the range of 0.341–0.558 for CRF, 0.201–0.474 for PD, 0.301–0.564 for SD, and 0.152–0.478 for QoL.

**Table 2:** Correlation analysis and partial correlation matrix.

	1	2	3	4	5	6	7	8	9	10	11	12	13	14	15	16	17	18	19	20
T1_CRF	1																			
T2_CRF	0.505**	1																		
T3_CRF	0.365**	0.558**	1																	
T4_CRF	.312**	.351**	.493**	1																
T5_CRF	.341**	.348**	.380**	.444**	1															
T1_PD	.265**	.270**	.226**	.142**	.097*	1														
T2_PD	.185**	.264**	.199**	.107*	0.067	.440**	1													
T3_PD	.154**	.221**	.277**	.184**	.191**	.284**	.442**	1												
T4_PD	.236**	.267**	.257**	.254**	.249**	.245**	.240**	.474**	1											
T5_PD	.206**	.233**	.225**	.201**	.275**	.201**	.264**	.345**	.474**	1										
T1_SD	.364**	.195**	.106*	.161**	.128**	.324**	.234**	.193**	.161**	.136**	1									
T2_SD	.266**	.269**	.161**	.131**	0.079	.216**	.267**	.218**	.192**	.172**	.564**	1								
T3_SD	.157**	.164**	.188**	.184**	.182**	.096*	.189**	.283**	.240**	.188**	.326**	.509**	1							
T4_SD	.142**	.105*	.156**	.165**	0.088	0.050	.133**	.193**	.242**	.196**	.255**	.332**	.454**	1						
T5_SD	.195**	.136**	.163**	.237**	.216**	0.070	.126**	.164**	.237**	.271**	.301**	.273**	.437**	.512**	1					
T1_QoL	− 0.101*	− 0.113*	− 0.096*	− 0.084	− 0.011	− 0.219**	− 0.182**	− 0.065	− 0.053	− 0.079	− 0.158**	− 0.148**	− 0.134**	− − 0.096*	− 0.153**	1				
T2_QoL	− 0.214**	− 0.252**	− 0.227**	− 0.134**	− 0.080	− 0.268**	− 0.286**	− 0.228**	− 0.191**	− 0.129**	− 0.221**	− 0.267**	− 0.130**	− 0.114*	− 0.113*	.371**	1			
T3_QoL	− 0.164**	− 0.279**	− 0.263**	− 0.216**	− 0.135**	− 0.219**	− 0.317**	− 0.254**	− 0.190**	− 0.168**	− 0.210**	− 0.331**	− 0.368**	− 0.241**	− 0.233**	.225**	.415**	1		
T4_QoL	− 0.150**	− 0.237**	− 0.289**	− 0.294**	− 0.222**	− 0.127**	− 0.223**	− 0.307**	− 0.355**	− 0.286**	− 0.132**	− 0.204**	− 0.344**	− − 0.334**	− 0.296**	.228**	.336**	.478**	1	
T5_QoL	− 0.182**	− 0.215**	− 0.211**	− 0.292**	− 0.204**	− 0.081	− 0.203**	− 0.167**	− 0.301**	− 0.251**	− 0.210**	− 0.169**	− 0.286**	− 0.308**	− 0.355**	.152**	.220**	.276**	.417**	1
M	2.175	2.587	2.941	2.687	2.375	7.520	7.218	6.560	6.133	6.042	1.744	1.948	2.075	1.846	1.532	2.408	2.051	1.975	2.115	2.253
SD	0.734	0.780	0.729	0.771	0.847	1.642	2.550	1.814	2.023	1.886	0.854	0.725	0.686	0.694	0.758	0.668	0.783	0.703	0.719	0.767

The triangle on the correlation matrix represents the Pearson correlation coefficient. The lower triangle of the correlation matrix represents first-order partial; correlation coefficient of each variable at time T1, T1_PD, T1_SD; T1_LQ is the control variable with partial correlation, so the unbiased correlation coefficient is estimated. T1 = within one week after initial cancer diagnosis, T2 = within one week after start of surgical treatment, T3 = within one week after the end of all chemotherapy courses, T4 = six months after the end of chemotherapy, T5 = 12 months after the end of chemotherapy.

*P < 0.05, ** *P* < 0.01.

### 3Latent growth model

Table 3 shows the fit indices of the linear and quadratic growth models. The nonlinear growth model has a statistically significant and better fit index than the linear growth model. Hence, there is a non-linear developmental relationship among CRF, PD, SD, and QoL. Table 4 shows the parameter estimation results of the nonlinear latent variable growth model.

**Table 3:** Fit indices of linear and quadratic latent variable growth models.

Model	Variable	χ^2^	df	RMSEA	CFI	TLI	SRMR
Linear growth model	Cancer-related fatigue	401.661	10	0.303	0.280	0.280	0.199
	Psychological distress	72.687	10	0.121	0.851	0.851	0.091
	Sleep disturbance	237.427	10	0.231	0.597	0.597	0.141
	Quality of life	174.782	10	0.196	0.533	0.533	0.177
Quadratic growth model	Cancer-related fatigue	41.476	6	0.118	0.935	0.891	0.047
	Psychological distress	30.062	6	0.097	0.943	0.905	0.035
	Sleep disturbance	20.721	6	0.076	0.974	0.956	0.034
	Quality of life	17.356	6	0.067	0.968	0.946	0.035

**Table 4:** Parameter estimation results of nonlinear latent growth model.

Variables	Coefficient			Variance			Covariance		
	Intercept	Slope	Quadratic	Intercept	Slope	Quadratic	I vs. S	I vs. Q	S vs. Q
Cancer-related fatigue	2.166***	0.649***	− 0.150***	0.407***	0.223***	0.012***	− 0.154***	0.026**	− 0.048***
Psychological distress	7.525***	− 0.583***	0.051**	2.493***	1.682***	0.082***	− 1.054**	0.148*	− 0.345***
Sleep disturbance	1.738***	0.345***	− 0.099***	0.580***	0.264***	0.012***	− 0.281***	0.046***	− 0.052***
Quality of life	2.393***	− 0.373***	0.087***	0.266***	0.196***	0.010***	− 0.097*	0.013	− 0.042***

I vs. S = Intercept vs. slope; I vs. Q = Intercept vs. quadratic; S vs. Q = Slope vs. slope.

*P < 0.05, **P < 0.01, ***P < 0.001.

The initial CRF level was statistically significant and greater than 0 (Intercept = 2.166, *P* < 0.001), and CRF showed an upward trend over the five surveys (slope = 0.649, *P* < 0.001), although the growth rate gradually weakened (quadratic = − 0.150, *P* < 0.001). The variance of the intercept, slope, and quadratic equation were all statistically significant and greater than 0, indicating statistically significant individual differences in the initial CRF level and its developmental speed and trend.

The initial PD level was significantly greater than 0 (intercept = 7.525, *P* < 0.001), and PD showed a significant weakening trend over the five surveys (slope = − 0.583, *P* < 0.001), although the weakening speed was gradually moderated (quadratic = 0.051, *P* < 0.01). The variances of the intercept, slope, and quadratic of PD were all significantly greater than 0 (*P* < 0.001), indicating statistically significant individual differences in the initial level, linear development trend, and rate of change.

The initial SD level was statistically significant and greater than 0 (Intercept = 0.580, *P* < 0.001), and SD showed an increasing trend (slope = 0.345, *P* < 0.001) during the five investigations, although the increase rate gradually dropped (*P* < 0.001, quadratic = − 0.099), indicating statistically significant differences in the initial level, linear development trend, and rate of change among individuals (*P* < 0.001).

The initial QoL level was statistically significant and greater than 0 (intercept = 2.393, *P* < 0.001), and the QoL showed a statistically significant weakening trend during the five surveys (slope = − 0.373, *P* < 0.001), although the weakening speed was gradually moderated (*P* < 0.001, quadratic = 0.087), indicating statistically significant differences in the initial level, linear development trend, and rate of change among individuals (*P* < 0.001).

### Parallel growth model

**Figure 3:**
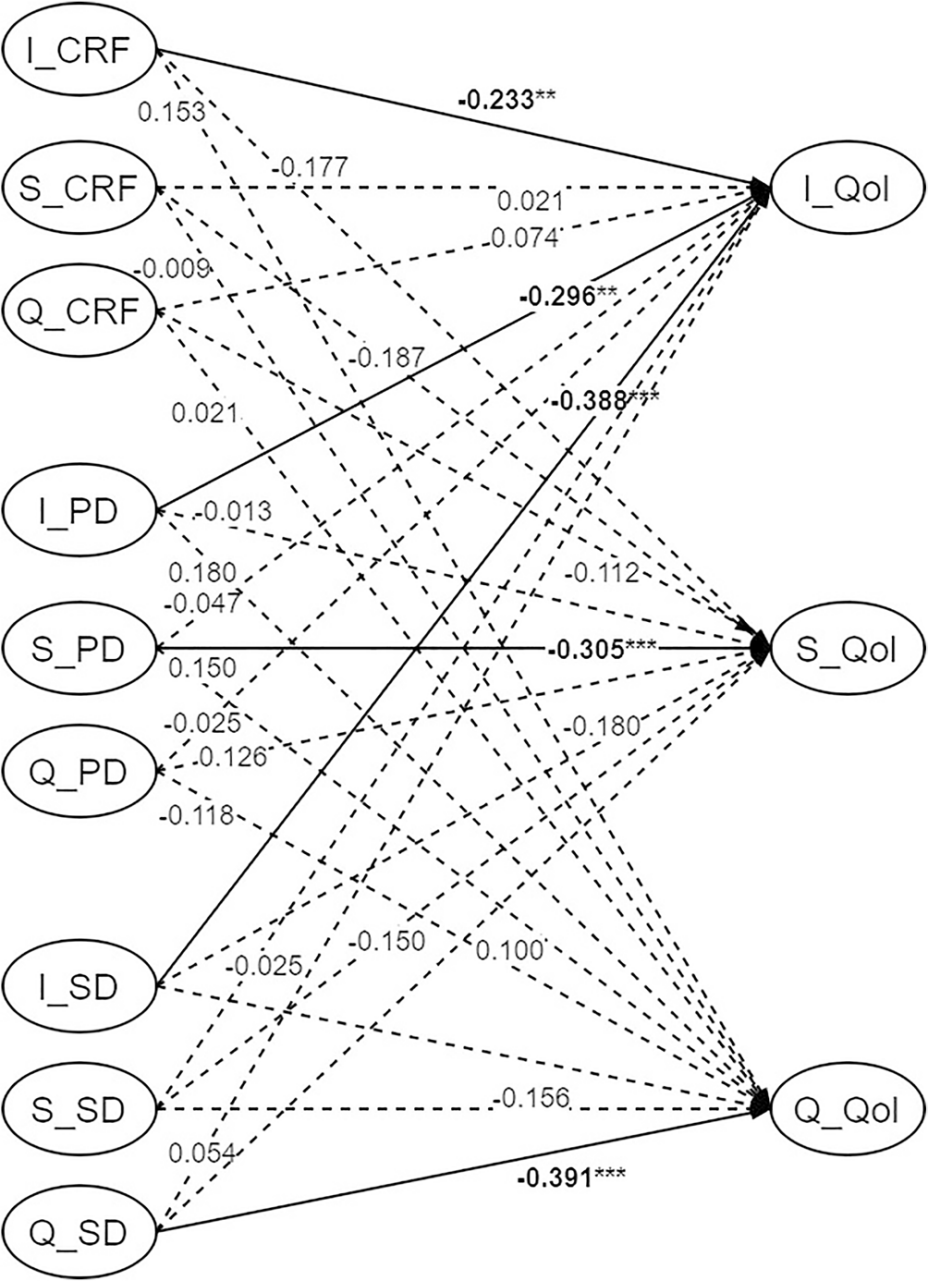
Parallel impact relationship model diagram. *Note* Significant paths are solid lines; Non-statistically significant paths are dashed; I = Intercept, S = Slope, Q = Quadratic, **P < 0.01; ***P < 0.001.

To explore the influence of CRF, PD, and SD on QoL, the parallel impact model of the growth factors among variables was set based on the quadratic growth model (Fig. 3). The model fit indices (χ^2^ = 244.873, df = 120, RMSEA = 0.049, CFI = 0.948, TLI = 0.918, SRMR = 0.038) all met the criteria. The initial levels of CRF (coefficient = − 0.233, *P* < 0.01), PD (coefficient = − 0.296, *P* < 0.01), and SD (coefficient = − 0.388, *P* < 0.001) each had a statistically significant negative effect on the initial level of QoL. The linear development rate of PD was statistically significant, and negatively affected the linear development rate of the QoL (coefficient = − 0.305, *P* < 0.05), and the quadratic development rate of SD negatively affected the quadratic development rate of QoL (coefficient = − 0.391, *P* < 0.05). The remaining relationship paths were not statistically significant (*P* > 0.05).

### Cross-lagged panel models

The cross-model fitting indices (χ^2^ = 267.883, df = 120, RMSEA = 0.054, CFI = 0.935, TLI = 0.900, SRMR = 0.075) showed that all model fit indices meet the standard criteria, indicating that the model is supported by the data and has a good structure (see in Supplementary Table 1).

The autoregressive path results show that prior moment CRF, PD, SD, and QoL have a significant positive effect on the autoregressive path of the subsequent moment (*P* < 0.001), indicating that each variable at the prior moment significantly promotes development at the next moment.

**Figure 4:**
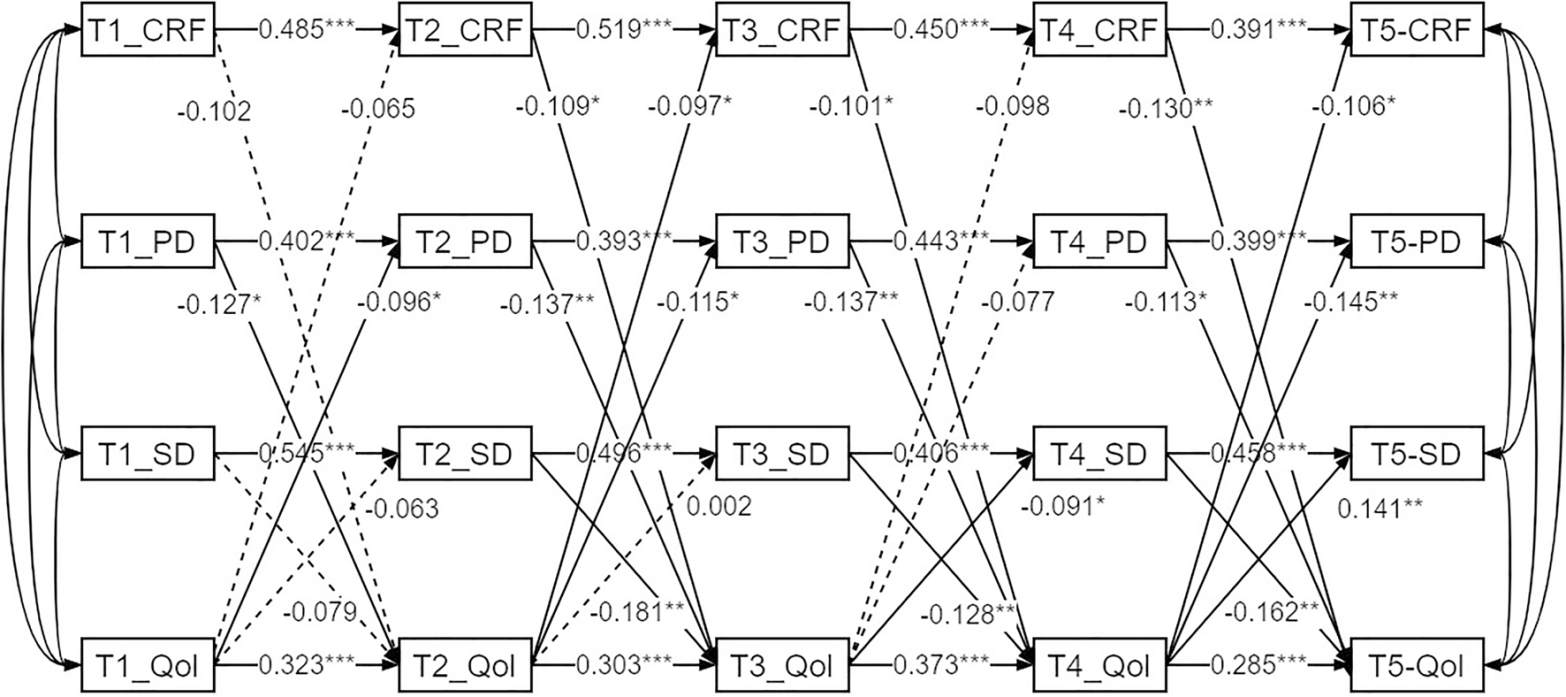
Cross-lag model path diagram. *Note* Significant paths are solid lines; Non-statistically significant paths are dashed; T1 = within one week after the initial cancer diagnosis, T2 = within one week after the start of surgical treatment, T3 = within one week after the end of all courses of chemotherapy, T4 = six months after the end of chemotherapy, T5 = 12 months after the end of chemotherapy; *P < 0.05; **P < 0.01; ***P < 0.001.

Cross-lagged regression path analysis result (Fig. 4) show that PD within one week after diagnosis (T1) has a significant negative effect on QoL within one week after surgery (T2) (*P* < 0.05, β = − 0.127). CRF, PD, and SD within one week after surgery (T2) have a significant negative impact on QoL within one week after all chemotherapy sessions (T3) (*P* < 0.05), with standardized coefficients of − 0.109, − 0.137, and − 0.181 respectively. CRF, PD, and SD within one week after chemotherapy (T3) have a significant negative impact on QoL within six months after chemotherapy (T4) (*P* < 0.05), with standardized coefficients of − 0.101, − 0.137, and − 0.128 respectively. CRF, PD, and SD within six months after chemotherapy (T4) have a significant negative impact on QoL within twelve months after chemotherapy (T5) (*P* < 0.05), with standardized coefficients of − 0.130, − 0.113, and − 0.162 respectively.

Additionally, QoL within one week after diagnosis (T1) has a significant negative effect on PD within one week after surgery (T2) (*P* < 0.05, β = − 0.096). QoL within one week after surgery (T2) has a significant negative effect on CRF and PD within one week after all chemotherapy sessions (T3) (*P* < 0.05), with standardized coefficients of − 0.097 and − 0.115 respectively. QoL within one week after chemotherapy (T3) has a significant negative effect on SD within six months after chemotherapy (T4) (*P* < 0.05, β = − 0.091). QoL within six months after chemotherapy (T4) has a significant negative effect on CRF, PD, and SD within twelve months after chemotherapy (T5) (*P* < 0.05), with standardized coefficients of − 0.106, − 0.145, and − 0.141 respectively. Other paths do not show significant effects between the various timepoints (*P* > 0.05).

## Discussion

The trajectory of CRF and SD changes is similar from T1 to T5. The CRF development trajectory shows an increasing trend and a gradually weakening growth trend. Similarly, the SD development trajectory shows an increasing trend and a gradually weakening growth trend. Within one week after the completion of chemotherapy, CRF and SD are most severe. A potential explanation for this is that when undergoing chemotherapy, along with cancer cells, some normal cells are destroyed, which reduces the body's resistance and causes patients to feel severe physical symptoms^12,24^. As time passes, the cancer-related fatigue and sleep disorders alleviate, but they persist for a long time. Fabi et al.^28^ showed that about 50% of patients still experience fatigue, even five years after cancer treatment, and 10-year cancer survivors have high levels of fatigue compared to the general population. As per the correlation analysis, cancer-related fatigue and sleep disorders are significantly positively correlated at all five measurement timepoints, consistent with Fox et al.’s^29^ results, indicating that CRF and SD are accompanied by development within 12 months after the completion of chemotherapy. From an individual perspective, there are individual differences in the initial levels and rate of change between five CRF and SD measurements. This may be because some individual internal variables regulate the self-perception of CRF and SD in different breast cancer patients. For example, Huang et al.^30^ showed that the younger and more educated breast cancer patients feel, the more serious CRF. This suggests that individual factors should be included in the in-depth investigation of CRF and SD formation and development mechanisms.

This study revealed that PD trajectories showed a decreasing trend, and the weakening trend is gradually moderated. Significant PD appears at the cancer diagnosis stage, reaching its peak. However, with the advancement of treatment, PD gradually decreases and reaches an adaptive state. This result is consistent with that of previous studies^31,32^ and shows that in a clinical situation, even if no special intervention is provided, patients facing stressors will gradually recover. This may be because of changes in patients’ resilience levels and emotion regulation^33,34^. The initial PD level has a statistically significant negative effect on initial QoL, indicating that the higher the initial PD, the lower the initial QoL. Furthermore, PD linear growth rate, which is statistically significant, negatively affects the QoL linear development rate, indicating that the faster the PD growth, the faster the QoL decline. Medical staff need to provide interventions such as conducting PD screening and assessment, and managing and providing psychological follow-up during early diagnosis to alleviate breast cancer patients’ high PD after diagnosis, thereby improving QoL^30,35^.

The initial CRF, SD, and PD levels significantly negatively affect initial QoL levels, suggesting that if patients already experience high CRF, SD, and PD levels at the time of new diagnosis, their QoL may significantly decrease. Therefore, medical staff should prioritize patients who have significant initial CRF, SD, and PD levels. Cross-lagged model analysis shows that CRF and SD measured at T2, T3, and T4 significantly negatively affect QoL measured at T3, T4, and T5, suggesting that surgery may be a stressor for physical discomfort symptoms. At T2, unpleasant post-surgery symptoms, such as CRF and SD directly affect changes in the next moment as the treatment progresses. PD measured at T1, T2, T3, and T4 significantly negatively affects QoL measured at T2, T3, T4, and T5, indicating that psychological discomfort begins to have a significant longitudinal impact on QoL as early as T1. At T1, PD experienced at the time of diagnosis directly affects changes in QoL at the next moment. Medical staff should adopt various strategies to reduce PD from T1 onwards, while intervening to alleviate CRF and improve SD from T2 onwards. This is of great significance for improving QoL.

This study was conducted during the COVID-19 pandemic, where breast cancer patients already faced issues like CRF, SD, and PD during treatment^36,37^. The spread of the COVID-19 virus undoubtedly aggravated these problems, further affecting the patients' QoL. First, COVID-19 infection can lead to a decrease in the body's immunity, worsening fatigue symptoms severity^38^. Patients might feel even weaker and more powerless. Second, although Rades et al.^39^ found that while COVID-19 appeared to have insignificant effects on sleep disorders in breast cancer patients, it is undeniable that symptoms caused by the virus, such as fever and cough, could disturb the patients' sleep. Moreover, PD is common among breast cancer patients during treatment. Breast cancer diagnosis and treatment themselves can exert immense psychological pressure on patients, and the COVID-19 virus threat adds to their fear and anxiety^40^. Patients might worry about the worsening of their condition and fear that it might not be curable, potentially further increasing their PD. At the same time, due to pandemic restrictions, patients might not be able to maintain normal social activities with family and friends, increasing their feelings of loneliness and psychological pressure^41^. Additionally, Baffert et al.^42^ found that the QoL for cancer patients was maintained and not affected during the COVID-19 pandemic. However, considering that CRF, SD, and PD interact with each other, they form a vicious cycle that significantly reduces the patients' QoL. Due to the impact of the pandemic, patients might not have been able to receive timely and effective medical services and support, further exacerbating their living difficulties. Although the follow-up was completed as planned, considering the significant impact of COVID-19, the results of this study should be treated with caution.

### Study limitations

First, the study variables were all assessed using questionnaire survey methods. Anonymous reporting was adopted to maximally ensure patients’ objectivity in the self-assessment of symptoms, but the questionnaire method itself suffers subjective bias that is difficult to overcome, and this may have affected patients’ willingness to answer. Future research should adopt implicit methods or combine physiological and neural indicators as measures in laboratory settings to obtain more objective and diverse data sources. Second, as COVID-19 put considerable pressure on patients, which will inevitably affect physical and psychological symptoms, such as causing patients to feel tired as well as experience sleep problems and negative emotions, future research should control for its effects. Furthermore, this study only analyzed the total scores of each scale, which may have resulted in the loss of specific information provided by subscales, thus failing to accurately reflect the actual performance of the assessed individuals in each dimension. Simultaneously, it is not possible to directly compare differences between different dimensions. It is recommended that future research explore the relationships between variables from a dimensional perspective.

## Conclusions

This study expands the literature on effects of common physical and psychological symptoms on QoL among breast cancer patients. These findings may help healthcare workers to better understand changes in SD, CRF, PD, and QoL, and their interactions in breast cancer patients, beginning from cancer diagnosis to one year after chemotherapy. The changing CRF and SD trends were consistent, showing an increasing trend that gradually weakened; the changing PD and QoL trends were consistent, both showing a weakening trend that was gradually moderated. Changes in each variable at a previous timepoint affected changes in them at the next timepoint, which suggests that intervention times should be moved forward. In particular, the PD development rate had statistically significant negative effects on the QoL development rate, suggesting that medical staff should provide interventions soon after cancer is diagnosed and actively take various nursing measures to alleviate high PD in breast cancer patients to improve their QoL.

### Supplementary Information

Supplementary Table 1.

## Data Availability

The datasets generated and/or analyzed during the current study is available from the corresponding author on reasonable request. Interested stakeholders may communicate with the corresponding author (Hong Chen) to access de-identified data sets.
