# Supplementary material for: Effects of sleep disturbance, cancer-related fatigue, and psychological distress on breast cancer patients’ quality of life: a prospective longitudinal observational study
Source: Sci Rep. 2024 Apr 15;14:8632. doi: 10.1038/s41598-024-59214-0 (PMC11018625; doi:10.1038/s41598-024-59214-0)
Supplement: Supplementary file 1 — Supplementary Table 1. [file 41598_2024_59214_MOESM1_ESM.docx]

**Supplementary table 1. Cross-lagged Model Test Results**

| Road | | | β | B | S.E. | t | P |
| --- | --- | --- | --- | --- | --- | --- | --- |
| **Autoregressive Effect (X_t_-X_t+1_)** | | | | | | | |
| T1_CRF | → | T2_CRF | 0.485 | 0.511 | 0.052 | 9.761 | <0.001 |
| T2_CRF | → | T3_CRF | 0.519 | 0.484 | 0.043 | 11.214 | <0.001 |
| T3_CRF | → | T4_CRF | 0.450 | 0.477 | 0.057 | 8.327 | <0.001 |
| T4_CRF | → | T5_CRF | 0.391 | 0.429 | 0.059 | 7.308 | <0.001 |
| T1_PD | → | T2_PD | 0.402 | 0.619 | 0.078 | 7.929 | <0.001 |
| T2_PD | → | T3_PD | 0.393 | 0.279 | 0.034 | 8.167 | <0.001 |
| T3_PD | → | T4_PD | 0.443 | 0.494 | 0.052 | 9.442 | <0.001 |
| T4_PD | → | T5_PD | 0.399 | 0.370 | 0.050 | 7.467 | <0.001 |
| T1_SD | → | T2_SD | 0.545 | 0.459 | 0.037 | 12.355 | <0.001 |
| T2_SD | → | T3_SD | 0.496 | 0.468 | 0.043 | 11.018 | <0.001 |
| T3_SD | → | T4_SD | 0.406 | 0.411 | 0.053 | 7.779 | <0.001 |
| T4_SD | → | T5_SD | 0.458 | 0.501 | 0.059 | 8.490 | <0.001 |
| T1_Qol | → | T2_Qol | 0.323 | 0.377 | 0.061 | 6.183 | <0.001 |
| T2_Qol | → | T3_Qol | 0.303 | 0.270 | 0.051 | 5.303 | <0.001 |
| T3_Qol | → | T4_Qol | 0.373 | 0.379 | 0.051 | 7.365 | <0.001 |
| T4_Qol | → | T5_Qol | 0.285 | 0.304 | 0.059 | 5.182 | <0.001 |
| **Cross-lagged Effect (X_t_-Y_t+1_)** | | | | | | | |
| T1_CRF | → | T2_Qol | -0.102 | -0.108 | 0.056 | -1.950 | 0.051 |
| T1_PD | → | T2_Qol | -0.127 | -0.060 | 0.025 | -2.417 | 0.016 |
| T1_SD | → | T2_Qol | -0.079 | -0.072 | 0.043 | -1.651 | 0.099 |
| T2_CRF | → | T3_Qol | -0.109 | -0.098 | 0.045 | -2.180 | 0.029 |
| T2_PD | → | T3_Qol | -0.137 | -0.038 | 0.013 | -2.838 | 0.005 |
| T2_SD | → | T3_Qol | -0.181 | -0.175 | 0.047 | -3.694 | <0.001 |
| T3_CRF | → | T4_Qol | -0.101 | -0.099 | 0.044 | -2.238 | 0.025 |
| T3_PD | → | T4_Qol | -0.137 | -0.054 | 0.018 | -2.932 | 0.003 |
| T3_SD | → | T4_Qol | -0.128 | -0.133 | 0.050 | -2.656 | 0.008 |
| T4_CRF | → | T5_Qol | -0.130 | -0.129 | 0.046 | -2.781 | 0.005 |
| T4_PD | → | T5_Qol | -0.113 | -0.043 | 0.018 | -2.326 | 0.020 |
| T4_SD | → | T5_Qol | -0.162 | -0.178 | 0.052 | -3.396 | 0.001 |
| **Reciprocal Effect (Y_t_-X_t+1_)** | | | | | | | |
| T1_Qol | → | T2_CRF | -0.065 | -0.075 | 0.051 | -1.472 | 0.141 |
| T2_Qol | → | T3_CRF | -0.097 | -0.090 | 0.039 | -2.282 | 0.023 |
| T3_Qol | → | T4_CRF | -0.098 | -0.107 | 0.056 | -1.916 | 0.055 |
| T4_Qol | → | T5_CRF | -0.106 | -0.126 | 0.059 | -2.151 | 0.031 |
| T1_Qol | → | T2_PD | -0.096 | -0.362 | 0.176 | -2.057 | 0.040 |
| T2_Qol | → | T3_PD | -0.115 | -0.266 | 0.115 | -2.314 | 0.021 |
| T3_Qol | → | T4_PD | -0.077 | -0.222 | 0.126 | -1.763 | 0.078 |
| T4_Qol | → | T5_PD | -0.145 | -0.381 | 0.126 | -3.028 | 0.002 |
| T1_Qol | → | T2_SD | -0.063 | -0.068 | 0.049 | -1.387 | 0.165 |
| T2_Qol | → | T3_SD | 0.002 | 0.002 | 0.040 | 0.048 | 0.962 |
| T3_Qol | → | T4_SD | -0.091 | -0.090 | 0.043 | -2.080 | 0.038 |
| T4_Qol | → | T5_SD | -0.141 | -0.151 | 0.050 | -3.028 | 0.002 |

Note: within one week after initial cancer diagnosis (T1), within one week after the start of surgical treatment (T2), within one week after the end of all courses of chemotherapy (T3), six months after the end of chemotherapy (T4), and 12 months after the end of chemotherapy (T5). β=Standardized coefficient, B=Unstandardized coefficient, S.E.=Standard Error.
